# Neurons in the barrel cortex turn into processing whisker and odor signals: a cellular mechanism for the storage and retrieval of associative signals

**DOI:** 10.3389/fncel.2015.00320

**Published:** 2015-08-21

**Authors:** Dangui Wang, Jun Zhao, Zilong Gao, Na Chen, Bo Wen, Wei Lu, Zhuofan Lei, Changfeng Chen, Yahui Liu, Jing Feng, Jin-Hui Wang

**Affiliations:** ^1^State Key Lab of Brain and Cognitive Science, Institute of Biophysics, Chinese Academy of SciencesBeijing, China; ^2^Department of Biology, University of Chinese Academy of SciencesBeijing, China; ^3^Department of Pharmacology and Collaborative Innovation, Center for Neurodegenerative Disorders in Shandong, Qingdao University, Medical CollegeDengzhou, China; ^4^Department of Physiology, Bengbu Medical CollegeBengbu, China

**Keywords:** conditioned reflex in mouse, learning, memory, neuron, astrocyte, barrel cortex, whisker and olfaction

## Abstract

Associative learning and memory are essential to logical thinking and cognition. How the neurons are recruited as associative memory cells to encode multiple input signals for their associated storage and distinguishable retrieval remains unclear. We studied this issue in the barrel cortex by *in vivo* two-photon calcium imaging, electrophysiology, and neural tracing in our mouse model that the simultaneous whisker and olfaction stimulations led to odorant-induced whisker motion. After this cross-modal reflex arose, the barrel and piriform cortices connected. More than 40% of barrel cortical neurons became to encode odor signal alongside whisker signal. Some of these neurons expressed distinct activity patterns in response to acquired odor signal and innate whisker signal, and others encoded similar pattern in response to these signals. In the meantime, certain barrel cortical astrocytes encoded odorant and whisker signals. After associative learning, the neurons and astrocytes in the sensory cortices are able to store the newly learnt signal (cross-modal memory) besides the innate signal (native-modal memory). Such associative memory cells distinguish the differences of these signals by programming different codes and signify the historical associations of these signals by similar codes in information retrievals.

## Introduction

Associative learning and memory are the bases of the cognitions (Byrne et al., 2007; Mayes et al., 2007; Suzuki, 2008; Lansner, 2009; Sanhueza and Lisman, 2013). Associative learning is a process in that experience and knowledge are acquired by the associations of two sensory signals or a sensory signal with a behavioral operation. The memories of these signals indicatively arise if they can be retrieved by cues. Two physiognomies of associative memory are the storage and distinguishable retrieval of these associated signals. In term of the cellular mechanisms for associative memory, activity-dependent plasticity at the synapses and neurons, e.g., long-term potentiation and depression, is presumably involved (Aou et al., 1992; Bliss and Collingridge, 1993; Alkon, 1998; Honey and Good, 2000; Blair et al., 2001; Christian and Thompson, 2003; Jones et al., 2003; Silva, 2003; Roman et al., 2004; Zhang et al., 2004; Dityatev and Bolshakov, 2005; Fanselow and Poulos, 2005; Weeks et al., 2007; Frey and Frey, 2008; Mozzachiodi et al., 2008; Neves et al., 2008; Nikitin et al., 2008; Sah et al., 2008; Wesson et al., 2008; Pape and Pare, 2010; Rosselet et al., 2011). Experience-dependent learning led to structural plasticity in spines and excitatory synapses (Trachtenberg et al., 2002; Sadaka et al., 2003; Holtmaat and Svoboda, 2009; Mégevand et al., 2009; Harlow et al., 2010; Wilbrecht et al., 2010; Ashby and Isaac, 2011; Cheetham et al., 2012; Margolis et al., 2012). The plasticity at the synapses and neurons indicates the end-point of associative memory, but does not reveal how these cellular units accept, memorize, and retrieve the associated signals. In other words, the neural plasticity does not signify the working principle that the neurons encode the storage and distinguishable retrieval of these associated signals. How the neurons are recruited to be associative memory cells that compute the associated signals for their storage remains to be addressed, especially *in vivo*, as memory processes are better to be examined *in vivo* (Hasegawa et al., 1998; Cadoret and Petrides, 2007; Won and Silva, 2008).

Conditioned reflex is used as a typical model of associative learning, in which the behaviors in response to the unconditioned stimulus can be evoked by the conditioned stimulus (Wasserman and Miller, 1997; Maren, 2008; Woodruff-Pak and Disterhoft, 2008). In this cross-modal reflex, the recall of the unconditioned signal is triggered by the conditioned signal and the cortex of encoding the unconditioned signal may become able to encode the conditioned signal (Wang et al., 2013, 2014). The associated signals to a given cortical area are the innate signal (unconditioned signal) and the newly acquired signal (conditioned signal). The storage of this newly learnt signal in this cortical area may need the recruitment of the neurons that do not encode any signal and the refinement of the neurons that encode the innate signal. How these neurons are recruited and refined to memorize the newly acquired and innate signals remains to be addressed. If the cortical neurons memorize multiple associated signals, how do the associative memory neurons distinguish their differences in information retrieval?

The neurons and glia cells presumably interact each other to fulfill brain functions (Schachner, 1991; Corty and Freeman, 2013). The molecules involved in neuron-astrocyte interactions may affect long-term memory (Florian et al., 2011; Suzuki et al., 2011). How the glia cells with the neurons are recruited to program the storage and retrieval of the associated signals during the conditioned reflex is unclear.

In the present study, we aim to reveal the principles that the cortical neurons and astrocytes are recruited to encode multiple signals for their associative storage and distinguishable retrieval. To address this question, we need an animal model of conditioned reflex, in which the cortical areas of encoding the sensory inputs are located on dorsal surface for easy access to the interested regions and for less injury to the cerebral circuits in subcortical nuclei. In addition, the cortical areas can form the connections for their respective innate signals to be associated and for one of them to be able to encode the associated signals. The barrel cortex meets these requirements since it is located at the dorsal surface of the cerebral cortex (Shepherd and Svoboda, 2005; Aronoff et al., 2010; Mao et al., 2011) and connects with the piriform cortex in cross-modal plasticity (Ye et al., 2012). The barrel cortex encodes whisker tactile sensation (Petersen, 2007) and the piriform cortex receives odor signal (Barkai and Saar, 2001; Wilson, 2001; Wilson and Sullivan, 2012). There is no subcortical connection between their afferent pathways (Haberly et al., 1987; Aronoff et al., 2010). The co-activations of the barrel and piriform cortices by pairing whisker and odor stimulations may induce their connections for them to encode the associated signals in cross-modal reflex. Based on these thoughts, we produced a novel mouse model of conditioned reflex (odorant-induced whisker motion), in which the barrel cortex was presumably the center.

In the control and odorant-induced whisker motion mice, our strategies are given below to reveal how the barrel cortical cells associatively memorize and distinguishably retrieve the new odor signal and innate whisker signal. The activities of multiple cells in associative memory are analyzed by two-photon calcium imaging and local field potential recording. How the individual neurons memorize and retrieve the associated signals is examined by intracellular recording and two-photon cell imaging to analyze their encoding patterns. If the neurons become able to encode the associated signals, the neurons memorize them. If the spatial and temporal activity patterns of these associative memory neurons are different in response to the associated signals, the neurons are able to distinguish their differences. The designation of whisker-dominant neurons or odor-dominant neurons is based on their activity strength and synchrony in response to signals, a principle similar to direction-selective cells in the visual cortex. The bases for the integrations of the associated signals are examined by tracing structural and functional connections between the barrel and piriform cortices.

## Methods and materials

All experiments were performed in accordance with the relevant guidelines and regulations by the Administration Office of Laboratory Animals at Beijing China. All experimental protocols were approved by Institutional Animal Care and Use Committee in the Administration Office of Laboratory Animals at Beijing China (B10831). The experimental conditions are listed in Table 1.

**Table 1:** **Experimental conditions are used in CR-formation and control mice**.

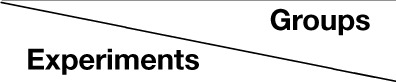		**Control mice**	**CR-formation mice**
Training procedures		NCG, in cage without either WS or OS. UPSG, in cage with WS and OS in the interval above 2–5 min, 5 times per day, 2 h in interval and 10 days	PSG, simultaneously pairing WS and OS in 20 s each time, 5 times per day, 2 h in interval and 10 days
Whisker stimuli (WS)		5 Hz mechanical vibrations to whiskers for 20 s	
Odor stimuli (OS)		Butyl acetate pulse toward the noses for 20 s	
Odor-test		Butyl acetate pulse toward the noses for 20 s	
Anesthesia		Urethane (1.5 g/kg)	
Temperature blanket set		37°C	
Recording location		Barrel cortex	
Cortical layers		LayerII-III	
Tracing dye		2 mM 1,1′dioctadecyl-3,3,3′,3-tetramethylindocarbocyanine perchlorate (DiI)	
Electrode resistance	LFP	5–7 MΩ	
	Intercellular recording	50–60 MΩ	
Pipette solution	LFP	150 NaCl, 3.5 KCl and 5 HEPES (mM)	
	Intercellular recording	2M KAc	
Fluorescence labeling		1 mM Oregon Green BAPTA-1-AM and 100 μM Sulforhodanmine-101	
ACSF		125 NaCl, 2.5 KCl, 26 NaHCO_3_, 1.25 NaH_2_PO_4_, 2 CaCl_2_, 1 MgCl_2_, and 20 glucose (mM) with pH 7.4 at 37°C	
Softwares for data analyses		Clampfit, NIH ImageJ, and MATLAB	
Statistical analyses		One-Way ANOVA is used for the comparison among multiple groups. Paired *t*-test is used for the comparisons in behavioral tasks before and after training, or in cellular responses to WS and OS	

### Mouse model of conditioned reflex (odorant-induced whisker motion)

**Figure 1:**
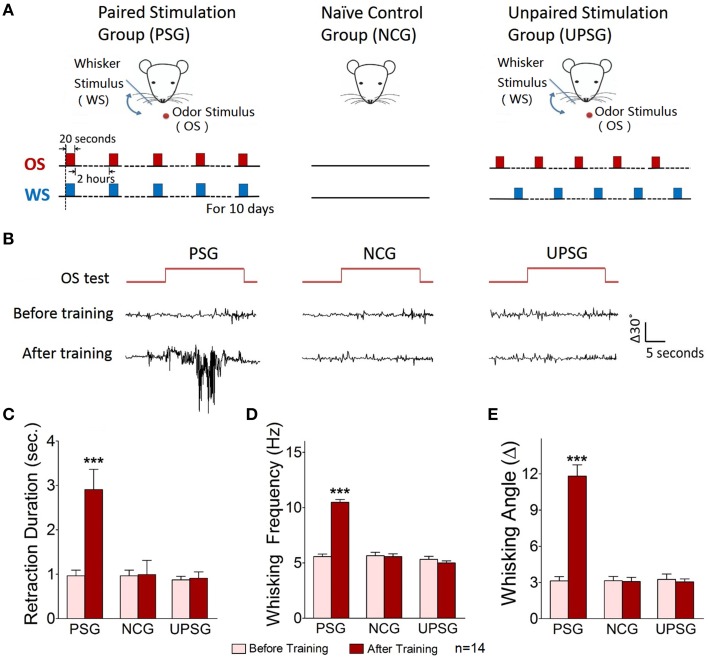
**A simultaneous pairing of whisker stimulus (WS) and olfactory stimulus (OS) leads to odorant-induced whisker motion**. Whisker stimulus (WS) was mechanical vibration pulses at 5 Hz in an intensity of evoking whisker fluctuation. Odor stimulus (OS) to the noses was butyl acetate pulse that sufficiently evoked olfactory bulb responses. The durations of both mechanical and odor pulses were 20 s. **(A)** Illustrates the procedures in pairing OS/WS stimulus group (PSG, left panel), naïve control group (NCG, middle), and unpaired stimulus group (UPSG, right). **(B)** Shows the responses of the trained whiskers to the odor-test (top red traces) before (middle black traces) and after training (bottom black) in PSG (left panel), NCG (middle), and UPSG (right). Calibration bars are 30° of whisker deflection and 5 s. **(C–E)** Show whisker retraction duration **(C)**, whisking frequency **(D)**, and whisking angle **(E)** in response to the odor-test before (light-red bars) and after trainings (dark-reds) in the PSG, NCG, and UPSG mice. The significant changes of these parameters are only seen in PSG mice (*p* < 0.001, *n* = 14 for each of groups; paired-test for a comparison before and after training; One-Way ANOVA for a comparison among groups). ^***^p < 0.001.

**Figure 2:**
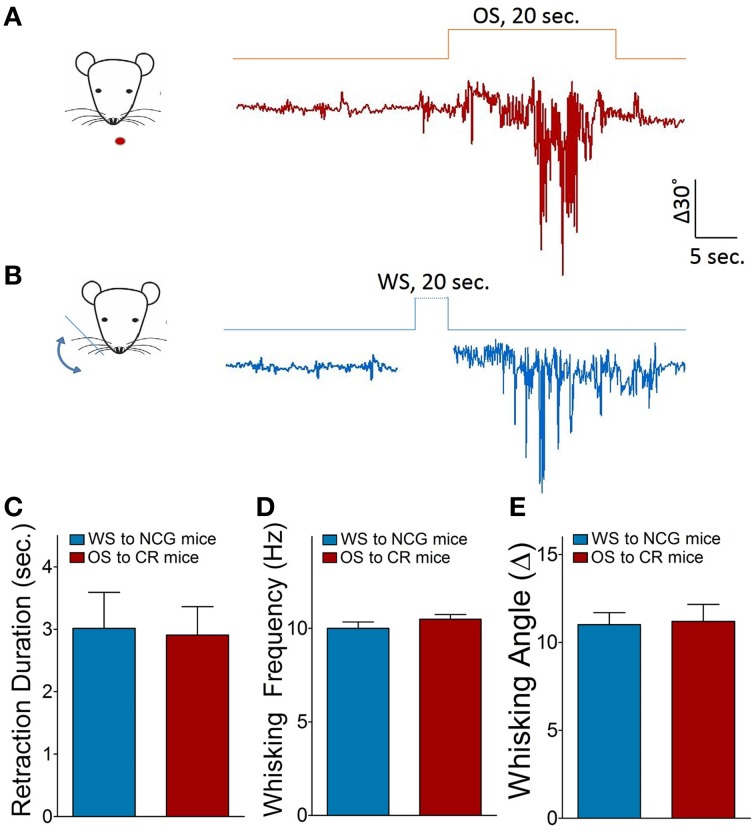
**Odorant-induced whisker motion is identified by seeing a similarity of whisker motion patterns induced by whisker and odor stimuli**. **(A)** Shows the pattern of whisker motion induced by odor stimulus in CR-formation mice. **(B)** Shows the pattern of whisker motion induced by whisker stimulus naturally in NCG mice. The patterns of whisker motions are similar in response to odor signal in CR-formation mice and in response to whisker signal in NCG mice. **(C–E)** Illustrates the comparisons of whisker retraction duration, whisking frequency and whisking angle induced by WS to NCG mice (blue bar) and by OS to CR-formation mice (red bar).

Strain C57 mice including males and females in postnatal day 20 were divided into three groups that received different treatments (Figure 1) in whisker stimulus (WS, 5 Hz mechanical stimulation) and odor stimulus (OS, butyl acetate). The trainings included a simultaneous pairing of conditioned OS with unconditioned WS (paired-stimulus group, PSG), WS/OS-unpairing (unpaired-stimulus group, UPSG; the interval between WS and OS about 2–5 min), and neither OS nor WS (naïve control group, NCG). WS and OS were given by the digital multiple sensory modal stimulator (MSMS) made in our laboratory. The OS was given by switching on butyl acetate-contained tube and generating a small liquid drop in front of the mouse noses (Video one in Supplementary Material). The intensity of butyl acetate odor was enough to induce the responses of olfactory bulb neurons detected by two-photon imaging (Figure S1). The WS to mouse assigned whiskers was given to the contralateral side of the barrel cortices that were studied in two-photon imaging and electrophysiology (Video one in Supplementary Material). The WS intensity suitably triggered whisker fluctuation (whisker-induced whisker motion, Figure 2). The parameters to train each mouse in PSG and UPSG by WS and OS were 20 s each training and five times per day in intervals of 2 h for 10 days (Figure 1). This training period was based on a fact that the onset of odorant-induced whisker motion reached the plateau level about 10 training days (**Figure 4D**). The stimulation intensity, duration and frequency were precisely controlled by MSMS, which were fixed for each trial and each mouse. During the training, each of the mice was placed in a home-made cage, in which their running and motion were restricted, but their body and arms freely extended. There are no circadian disturbance and stress conditions, such as noise, light, unusual odors, and motions from the experimenters. The mice were placed into the cage for 10 min every day about 1 week to have them habituated to experiment condition before the training, and placed into the cages about 5 min prior to each training for their quiet adaptation during the training. Care was also used in the odor-test procedure (please see below). It is noteworthy that the mice in NCG were placed in these home-made cages, but did not receive WS and OS.

The mouse whisker motion tracks were monitored by a digital video camera (SONY HDR-XR-550). All images were digitized (50 Hz) and converted into whisker motion traces. The whisker motions were quantified by public software (MB-Ruler, v5.0 by Markus Bader, MB-Software solution, Germany), including whisker retraction time, whisking frequency, and fluctuation magnitude. Whisker retraction was defined as backward motion >5° away from original position and 0.5 s. Whisker fluctuation magnitudes were defined as the absolute changes of whisking angles (Ni et al., 2010). The response of mouse whiskers to the odor-test (butyl acetate toward the noses for 20 s, Figure 1) was recorded before the training and 1 h after the end of each training day up to day 10 (**Figure 4D**) to quantify the onset time and level of odorant-induced whisker motion (conditioned reflex, CR). Odorant-induced whisker motion was accepted if the whisker motion met the following criteria. The patterns of odor-induced whisker motion were similar to typical whisker motions induced by WS (Figure 2), but not spontaneous whisking at low magnitudes. The whisking frequency, whisking angle, and whisker retraction time increased significantly, compared with control and before the training. This conditioned OS induced whisker motion that was originally induced by unconditioned WS, in which the odor signal induced the recall of whisker signal and led to whisker motion, i.e., CR-formation (Video two in Supplementary Material). It is noteworthy that odorant-induced whisker motion is not related to mouse sniffing, since the sniffing alters the baseline of whisker motion trace, which is not a case in our analyses (Figure 1). Whisking frequency is also greater than the sniffing, and all of the mice do not show the sniffing induced by OS-test.

The “assigned whiskers” were long whiskers (such as arcs 1–2) on the same side and same rows that were assigned for the training by mechanical whisker stimuli in the PSG and UPSG as well as for the odor-test in all mice. Their corresponding barrels were studied in field potential recording, intracellular recording and two-photon cell imaging. We did not trim short whiskers since the whisker trimming raised the excitability of the barrel cortices (Zhang et al., 2013), which might affect an onset of conditioned reflex.

To test CR-formation in the barrel cortex, we used an approach to silence this region by injecting 6-Cyano-7-nitroquinoxaline-2,3-(1H,4H)-dione (CNQX) and D-amino-5-phosphonovanolenic acid (D-AP5) into the barrel cortex with the glass pipettes (Matyas et al., 2010; O'Connor et al., 2010) to inhibit excitatory synapses (Zhang et al., 2013). If the associated signals were integrated in the barrel cortex for CR-formation, the silence of the barrel cortex should block odorant-induced whisker motion. Before and after using CNQX and D-AP5, odorant-induced whisker motion and whisker-induced whisker motion were examined.

### Electrophysiological recording

The mice were anesthetized by the intraperitoneal injections of urethane (1.5 g/kg). In surgical operation, anesthetic depth was set as lack of reflexes in pinch withdrawal and eyelid blinking. Body temperature was maintained by using a computer-controlled heating blanket at 37°C. The barrel cortices were located based on the distribution of surface vessels (Zhao et al., 2012), the map of the mouse brain (Paxinos and Franklin, 2001) and their responses to whisker stimuli, which were confirmed by histology after each experiment. A craniotomy (2 mm in diameter) was made on the skull above the center of barrel cortex about 1 mm posterior to the bregma and 3.0–3.5 mm lateral to midline. The anesthetic depth for the mice during electrophysiological study *in vivo* was set at moderate reflexes of pinch withdrawal and eyelid blinking as well as the responses of their whiskers to stimulations, i.e., light anesthesia from their partial recovery of surgical anesthesia.

Local field potentials (LFP) were recorded in layers II–III of the barrel cortices by glass pipettes that contained standard pipette solution (150 mM NaCl, 3.5 mM KCl, and 5 mM HEPES). The resistance of the recording pipettes was 5–7 MΩ. Electrical signals were inputted to an AxoClamp-2B amplifier and pClamp 10 (Axon Instrument Inc. CA USA) for data acquisition and analysis. The electrical signals were digitized at 10 kHz and filtered by low-pass at 0.5 KHz. In data analyses, the band-pass filter (1–100 Hz) and the second order “Savitzky–Golay” filter were used to isolate LFP signals. LFP signals were complex and variable. Individual LFP events induced by WS or OS lasted for 10 ms with a sharp negative response. The differences between negative peaks and baseline in individual LFPs were measured and averaged to show stimulus-evoked LFP amplitude. LFP frequency was calculated as one over inter-event intervals.

The intracellular recording of synaptic activity and neuronal spikes was conducted in layers II–III of barrel cortex by sharp electrodes that contained standard pipette solution (2 M KAc). The resistance of the recording electrodes was 50–60 MΩ. Electrical signals were inputted to AxoClamp-2B amplifier and pClamp 10 system for data acquisition and analyses. The signals were digitized at 20 kHz and filtered by low-pass (3 KHz). In the analyses of synaptic integrated potentials and spikes, inter-event intervals were measured to present their frequencies that equaled to one over inter-event intervals. It is noteworthy that the recordings of LFP, intracellular signals, and two-photon Ca^2+^ signals were done in the identical regions of the barrel cortices (Zhao et al., 2012).

In electrophysiological recordings, the test stimulations by odorant and whiskers' deflection were given to the mice. The odor-test to the noses or the mechanical pulses to the whiskers on the contralateral side of the recorded cortical areas were given to induce neuron responses in the barrel cortices, in which the parameters of the stimulus intensity, frequency, and duration were consistent with those in behavioral trainings. In the sequential WS and odor stimulus, inter-pulse intervals were 20 s.

### Fluorescence labeling

The mice were anesthetized and surgically operated by methods similar to those in electrophysiology section. The dura was intact except for a few tiny holes made by glass pipette for dye injections. The injuries to the cerebral cortices and surface vessels were avoided (Zhao et al., 2012). Ca^2+^ dye, Oregon Green BAPTA-1-AM (OGB-1, Invitrogen USA), was applied to monitor the activities of the cortical neurons and astrocytes. OGB-1 was dissolved in DMSO and 20% Pluronic F-127 (Invitrogen, USA) for stock solution at 10 mM. This stock solution was diluted in the ACSF to yield final concentration at 1 mM, which was injected into layer I–II of the barrel cortices by the pressure (1 bar, 5 min) through glass pipettes (100 μm below the pia) to label the multiple cells. In the meantime, 100 μM sulforhodanmine-101 (SR101, Invitrogen) was co-injected to label the astrocytes (Zhao et al., 2012). The volumes of the dyes were controlled at −0.5 μl. After the injections, a craniotomy well was filled by low-melted agarose (1%) in the ACSF and sealed with a glass cover-slip. The exposed skull was adhered to a custom-made metal recording chamber with dental acrylic cement and superfused with the ACSF (in mM): 125 NaCl, 2.5 KCl, 26 NaHCO_3_, 1.25 NaH_2_PO_4_, 2 CaCl_2_, 1 MgCl_2_ and 20 glucose (pH 7.4) at 37°C and bubbled with 95%O_2_/5% CO_2_ (Zhang et al., 2012).

### Two-photon cell imaging

The calcium imaging was done at the neurons and astrocytes of layers II–III in the barrel cortex 1 h after dye injections under a confocal scanning microscope (Olympus FV1000, Tokyo, Japan) equipped with a two-photon laser-beam generator (Mai Tai, Physical Spectrum, USA). They were mounted to an upright microscope (Olympus BX61WI) with water immersion objective (40X, 0.8NA). The two-photon laser beam (810 nm) was given to excite OGB and SR101. The average power delivered to the barrel cortices was < 75 mW. Emission wavelengths were 523 nm for Ca^2+^-binding OGB and 603 nm for SR-101. Whole field images were acquired at 10 Hz frame rate (256 × 256 pixels). The parameters set for the laser beam and photomultiplier tube were locked for the measurements throughout all experiments to maintain consistent conditions in comparisons among groups. OGB-labeled cells were those cells detected by this two-photon microscope. In addition, the anesthetic depth of mice in the imaging study was set at moderate reflexes (please see electrophysiology section).

The activity patterns of the barrel cortical neurons and astrocytes in response to OS and WS were measured *in vivo*. Cellular responses were induced by the odor-test to the noses and the mechanical pulses to the whiskers on the contralateral side of the recorded barrel cortices, in which the stimulus parameters were consistent with those in the behavior training. The stimulations of olfaction and whiskers were pair-pulses (OS vs. WS or turned around) with 30 s of intervals. The magnitude of intracellular Ca^2+^ signals was positively correlated to spike frequency, and the duration of Ca^2+^ signals was correlated with spike number. So, Ca^2+^ levels in a neuron indicated its response strength *in vivo* (Petersen et al., 2005; Yaksi and Friedrich, 2006; Moreaux and Laurent, 2007). The activity of the astrocytes also altered their Ca^2+^ signals (Halassa et al., 2007). The synchrony of Ca^2+^ signals among cell pairs was analyzed by correlation coefficients to represent their activity synchrony (Hirase et al., 2004; Takata and Hirase, 2008; Golshani et al., 2009).

### Imaging data analyses

Cellular Ca^2+^ fluorescence signals in response to stimuli were acquired by Fluoviewer-10 software (Olympus Inc. Japan) and analyzed in cell bodies by NIH ImageJ and MATLAB (MathWorks). Ca^2+^ signals from each cell were analyzed by marking circles on their somata (a region of interest, ROI). To reduce photon and PMT noises, a median filter (radius, 1 pixel) was used to all images. Ca^2+^ fluorescence signals in cell responses were digitized as signal traces, and then were normalized and presented as relative fluorescence change (ΔF/F; Zhao et al., 2012). Baseline fluorescence (F) was an averaged value in the ROI before stimuli. ΔF values were the differences between the evoked cell Ca^2+^ signals and the baseline. Fluorescence signals were also subtracted from noise signals of unstained blood vessels (Zhao et al., 2012). The normalized Ca^2+^ signals were smoothed by a low-pass Butterworth filter to remove low-level fluctuation and minimize distortion from fast Ca^2+^ transients (Moreaux and Laurent, 2007). The effective Ca^2+^ signals from active cells were judged based on a criterion that ΔF/F was >2.5 times of the standard deviation of baseline values lasting for 500 ms.

The pairwise cross-correlation of normalized and smoothed Ca^2+^ signals (ΔF/F) in the pairs of the neurons or the astrocytes was analyzed based on Pearson's correlation (Takata and Hirase, 2008; Golshani et al., 2009). Although, the cross-correlations in neuron-pairs were higher from raw fluorescence traces than deconvolution traces over two-folds (Smith et al., 2010; Smith and Haüsser, 2010), we computed the raw traces without temporal deconvolution in neurons consistently with those in astrocytes (Nedergaard et al., 2010; Zhang et al., 2012). Considering two signals *x*(*t*) and *y*(*t*) of real variable t; the cross-correlation *r* at delay *d* is defined as:

(1) $$\text{r}=\frac{\sum_{t}\left[\left(x(t)-mx)\times (y(t-d)-my\right)\right]}{\sqrt{\sum_{t}{\left(x(t)-mx\right)}^{2}}\times \sqrt{\sum_{t}{\left(y(t-d)-my\right)}^{2}}},$$

*mx* and *my* are the means of the corresponding series. The correlation coefficients normalized to the autocorrelation at zero lag were calculated. Based on the calculations, the correlation matrices were plotted using MATLAB 7.0. The data were presented as mean ± SEM.

It is noteworthy that neuronal activity levels are defined as the frequency and amplitude of field potential, the frequency of spikes and synaptic potentials in intracellular recording, as well as the response strength of calcium signals in two-photon cell image. Activity level and activity synchrony are called as the spatial and temporal patterns of neuronal activities. The correlations among the cells might be negative, however, we used their absolute values in our data presentation. The larger the correlation value, the better the synchrony among these cells, or vice versa. In the analyses of the neurons to encode odor and whisker signals by two-photon imaging and intracellular recording, if the neurons become processing the associated signals, the neurons memorize these signals, similar to memory B-cells in immune responses. If the spatial and temporal activities of these neurons are different in response to these associated signals, the neurons are able to distinguish the difference of these signals, similar to direction-selective cells in the visual cortex.

### Identification of neural connections

The connection between the barrel and piriform cortices was idenfied by neural tracing (Zhang et al., 2013). In morphologic tracing, we used a lipophilic reagent 1,1′dioctadecyl-3,3,3′,3-tetramethylindocarbocyanine perchlorate (DiI; Beyotime China). The membrane permeable DiI diffuses in the cell and on the membrane, which is used as both anterogarde and retrograde tracings. 2 mM DiI in DMSO was injected into the barrel cortices. The animal surgery and injection location were similar to those of injecting calcium dye. After DiI injection, the injection area was sealed and the surgery area healed. After 24 h, the mice were anesthetized by the intraperitoneal injections of sodium pentobarbital, and were perfused by 4% paraformaldehyde in 0.1 M phosphate buffer solution into left ventricle/aorta until their bodies were rigid. The brains were quickly isolated and fixed in 4% paraformaldehyde PBS for additional 24 h. Mouse cortical tissues were sliced in the coronal sections of the brain including the barrel cortex at 100 μm by a Vibratome from anterior to posterior. The slices in series collection were placed under the confocal laser scanning microscope, where excitation wavelength was 549 nm, emission wavelength was 565 nm, and PMT sensitivity was set consistently. Axon projection and cell labeling were traced between the barrel and piriform cortices in the single slices.

In addition, we examined functional connection between the barrel and piriform cortices *in vivo*, in which the electrical stimuli (0.2 ms) were given in the barrel cortex by two-polar tungsten electrodes and LFP was recorded in the piriform cortex by the glass pipettes that contained standard pipette solution (150 mM NaCl, 3.5 mM KCl, and 5 mM HEPES; please see the section of electrophysiological recording). The intensities and duration of electrical stimuli were set to be identical for the brains in the mice of CR-formation and NCG. The functional connections between them were also examined in brain slices (Figures 2G–I) by recording LFPs in layers II–III of the barrel cortex with the glass pipettes and by electrically stimulating layers III of the piriform cortex (please see above).

There are a few rules for analyzing LFP. (1) In a given recording site, population EPSPs point in the opposite direction of population spikes since they generate in different loci, such as EPSPs at dendritic synapses and spikes at soma/axon hillock. (2) The durations of population EPSPs are longer than those of population spikes, as EPSPs are >20 ms and spikes are < 2 ms in the individual neurons. (3) As orthodromic spikes are triggered by EPSPs, the orthodromic population spikes are onset in orthodromic population EPSPs. So, the onset of population spikes follows the onset of population EPSPs, the time of population spikes fall into the period of population EPSPs and their waveforms have opposite directions. (4) The onset of antidromic population spikes is earlier than that of orthodromic population EPSPs, as the time for the propagation of antidromic spikes on the axons is shorter than the time delay of synaptic transmission.

### Statistical analysis

Paired *t*-test was used in the comparisons of the experimental data before and after associative learning as well as the neuronal responses to WS and OS in each of the mice. One-Way ANOVA was used to compare the changes of neuronal activity and morphological quantification between the controls and associative learning groups.

## Results

### Pairing stimulations to whiskers and olfaction leads to odorant-induced whisker motion

Mice were divided into three groups to receive the simultaneous pairing of unconditioned WS and conditioned odorant stimulus (OS; paired-stimulus group, PSG), the unpairing of WS and OS (unpaired-stimulus group, UPSG) or no stimulations (naïve control group, NCG; Figure 1). The procedure consisted of each training for 20 s, five times with 2 h interval per day and 10 days. Odorant-induced whisker motion was onset if its whisking pattern had no statistical difference from whisker-induced whisker motion (Figure 2; *p*>0.2, *n* = 14; One-Way ANOVA).

Odorant-induced whisker motion is onset after pairing WS and OS. Figure 1B show the responses of the assigned whiskers to the odor-test (top-red pulses) in PSG, NCG, and UPSG mice. By comparing whisker motions before (black traces in middle) and after the training (bottom), we see that the odor-test induces whisker motions in PSG mice, but not UPSG and NCG. Whisker retraction duration (Figure 1C), whisking frequency (Figure 1D), and angles (Figure 1E) in response to the odor-test are different before (light-red bars) and after OS/WS-pairing (red) in PSG mice (*p* < 0.001, *n* = 14; One-Way ANOVA), but not in UPSG (*n* = 14) and NCG (*n* = 14). The associations of whisker and odor signals lead to odorant-induced whisker motion, a new type of conditioned reflex (CR). PSG mice with CR are named as CR-formation mice.

To study cellular mechanisms underlying the associative storage and distinguishable retrieval of multiple signals, we used NCG and CR-formation mice. The rationale for not including UPSG was based on the facts that UPSG mice did not express odorant-induced whisker motion (Figure 1) and their barrel cortical neurons did not respond to butyl acetate (**Figure 5**).

### An association of whisker and odor signals induces connection between barrel and piriform cortices

**Figure 3:**
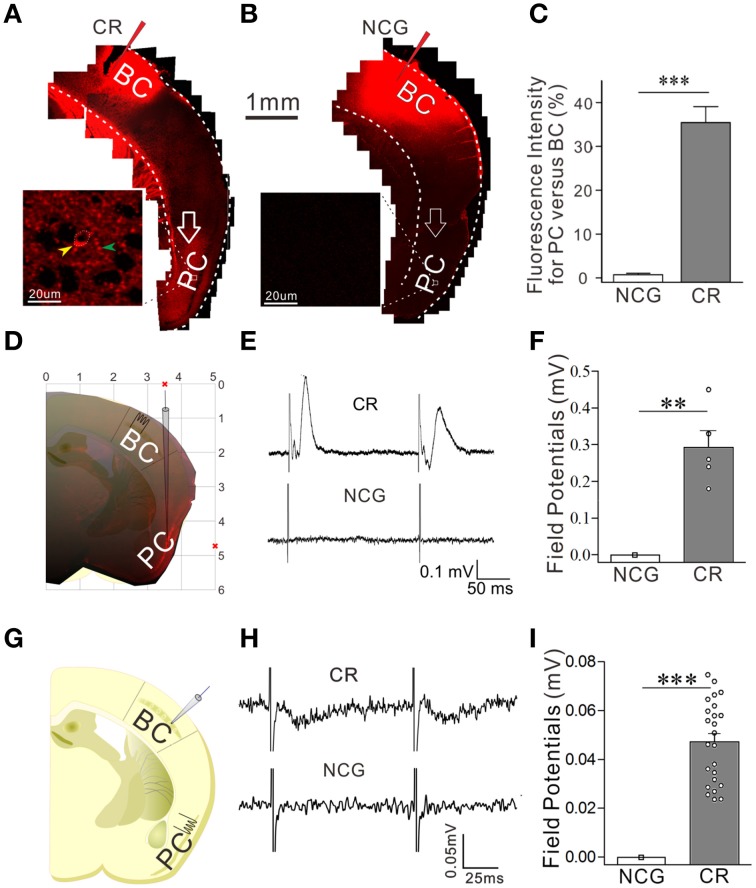
**The connection between the barrel and piriform cortices is established after associative learning**. The structural connection was traced by injecting 1,1′dioctadecyl-3,3,3′,3-tetramethylindocarbocyanine perchlorate into the barrel cortex and seeing its presence in the piriform cortex. The functional connection was examined by recording LFP in the piriform cortex and electrically stimulating the barrel cortex *in vivo*, or turned around. In *in vivo* recordings, bipolar tungsten electrodes were placed in barrel cortex, and glass recording electrodes (10 MΩ) were positioned into the piriform cortex (0.34–0.58 mm posterior to the bregma, 3.25–3.5 mm lateral to midline, and 4.75–5.0 mm in depth). **(A)** Shows neural tracing from the barrel cortex to the piriform cortex in CR-formation mouse. An arrow points fluorescent labeling in the piriform cortex. Left panel shows an enlarged image from the piriform cortex which includes DiI-labeled neuron (yellow arrow) and DiI-labeled axons (green arrow). **(B)** Shows the neural tracing from the barrel cortex to the piriform cortex in a NCG mouse. An arrows indicates no fluorescent labeling in the piriform cortex. **(C)** Shows the comparison of neural tracing in the piriform cortex from CR-formation mice (*n* = 9, gray bar) and NCG mice (*n* = 9, white), based on relative fluorescent intensity. **(D)** Shows LFP recording in the piriform cortex by a glass pipette of including DiI and the electrical stimulation in the barrel cortex *in vivo*. **(E)** Top trace shows LFP in the piriform cortex recorded from a CR-formation mouse and bottom trace shows no LFP recorded in the piriform cortex from a NCG mouse. **(F)** Illustrates the comparison of LFPs recorded in the piriform cortex from CR-formation group (*n* = 5 recordings from three mice, gray bar) and NCG (*n* = 6 recordings from three mice, white bar). **(G)** Shows LFP recording in the barrel cortex and electrical stimuli in the piriform cortex in the brain slices. **(H)** Top trace shows LFP in the barrel cortex recorded from a CR-formation mouse, and bottom trace shows no LFP recorded in the barrel cortex from a NCG mouse. **(I)** Illustrates the comparisons of electrical signals recorded in the barrel cortex from CR-formation mice (*n* = 23 recordings from five mice, gray bar) and NCG mice (from five mice, white). ^**^p < 0.01; ^***^p < 0.001.

Odorant-induced whisker motion may be based on the formation of axon connections between the barrel and piriform cortices, as the wiring is detected in cross-modal plasticity (Ye et al., 2012). The structural connections between the barrel and piriform cortices were traced by injecting 1,1′dioctadecyl-3,3,3′,3-tetramethylindocarbocyanine perchlorate (DiI) in the barrel cortices. Compared to neural tracing in controls (Figures 3B,C, *n* = 9), DiI is detected in the piriform cortex and layer-VI white matter in CR-formation mice (Figures 3A–C, *p* < 0.001, *n* = 9; One-Way ANOVA). As DiI is detected in axonal terminals and cell bodies of the piriform cortex (enlarged images in Figures 3A,B), the mutual innervation between the barrel and piriform cortices forms after associative memory. It is noteworthy that the connection may not form through the intermediate brain areas since DiI is not trans-synaptic dye and digital spikes do not cross over chemical synapses.

The functional connection was examined by recording LFP in the piriform cortex and stimulating the barrel cortex *in vivo*, or turned around. Electrical stimulus to the barrel cortex induces synaptic responses and neuronal spikes in the piriform cortex of CR-formation mice (top trace in Figure 3E and gray bar in Figure 3F, *n* = 5 recordings from three mice), but not control mice (*n* = 6 recordings from three mice, *p* < 0.01; One-Way ANOVA). Moreover, electrical stimulus to the piriform cortex induces field potentials at the barrel cortices in brain slices from CR-formation mice (top trace in Figure 3H and gray bar in Figure 3I, *n* = 23 recordings from five mice), but not controls (from five mice, *p* < 0.001; One-Way ANOVA). These results confirm that the mutual innervations between the barrel and piriform cortices are functional. In addition to new connections, their fucntional connections may need the upregulation of axon functions (such as axon transportation and spike propagation) and/or the conversion of inactive synapses into active ones.

**Figure 4:**
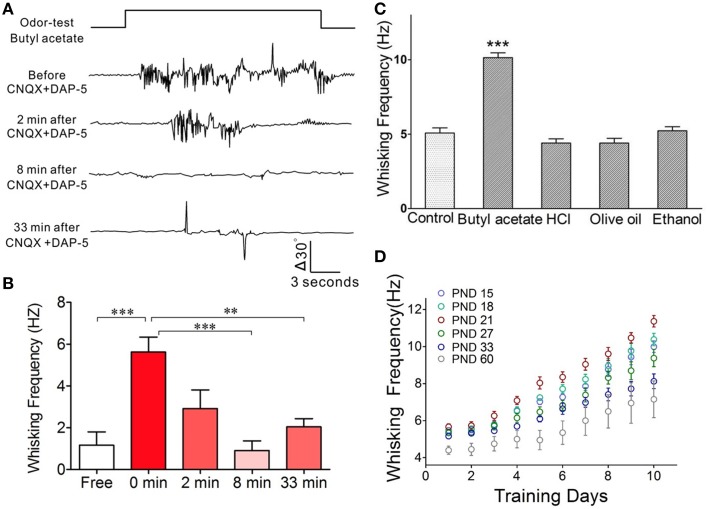
**The barrel cortex is essential to odorant-induced whisker motion that is an odorant specific**. **(A)** The blockade of neural activities in the barrel cortex removes odorant-induced whisker motion. In CR-formation mice, excitatory synaptic transmission in the barrel cortex was blocked by locally injecting CNQX/DAP-5 (400/500 μM), and neuronal spikes were blocked by locally injecting TTX (20 μM). The odor-test pulse was given toward the noses, and odorant-induced whisker motion was monitored by a digital video camera. Traces from the top to bottom show whisker motion tracks induced by the odor-test (top trace) toward the noses in a CR-formation mouse before and after injecting CNQX/DAP-5 for 2, 8, and 33 min. Calibration bars show whisker motion angle and time. **(B)** Shows whisking frequencies in CR-formation mice (*n* = 5) before and after injecting these reagents (*p* < 0.001) as well as for 0 vs. 8 and 33 min (*p* < 0.01; One-Way ANOVA). **(C)** In CR-formation mice trained by pairing WS and OS (butyl acetate), the whisker motions are examined by giving different odorants, such as butyl acetate (10%), hydrochloric acid (5%), olive oil (100%), and ethanol (75%). Odorant-induced whisker motion is seen by giving the test of butyl acetate only. Whisking frequency is increased by giving butyl acetate test (dark-gray bar), compared with that before WS/OS-pairing (light-gray; *p* < 0.05, *n* = 5; One-Way ANOVA), but not by the tests of hydrochloric acid, olive oil and ethanol (dark-gray). **(D)** Illustrates frequency in odorant-induced whisker motion vs. days in pairing OS and WS in different ages of the mice (postnatal days, PND 15, 18, 21, 27, 33, and 60; *n* = 12 for each of groups). The optimal age of the mice who are trained to express odorant-induced whisker motion is PND 21 when a maximal level of whisking frequency is seen. ^**^p < 0.01; ^***^p < 0.001.

In terms of cellular mechanisms underlying this associative memory, we examined how the barrel cortical neurons and astrocytes process these associative signals in CR-formation mice. The rationale for studying the role of neurons and astrocytes in associative memory is based on the reports that the neuron-astrocyte interaction may affect long-term memory (Florian et al., 2011; Suzuki et al., 2011). The rationale for studying the barrel cortex instead of the motor cortex is based on our result that the inhibition of synaptic and neuronal activities in the barrel cortices removes odorant-induced whisker motion (Figures 4A,B; *p* < 0.001, *n* = 5; One-Way ANOVA). The barrel cortex becomes the primary center of this conditioned reflex. The rationale for using butyl acetate as odor test, but not others, is based on the odorant specificity of odorant-induced whisker motion that in CR-formation mice is evoked by butyl acetate, but not olive oil, hydrochloric acid, and ethanol (Figure 4C; *p* < 0.01, *n* = 5; One-Way ANOVA). Moreover, the frequencies of odorant-induced whisker motion reach the maximal level in the mice that are trained starting at postnatal day 21 (Figure 4D) which is the rationale for us to train the mice starting at postnatal day 20.

### Neurons and astrocytes in the barrel cortex process whisker and odor signals after association

In the investigation that the barrel cortex encoded odor and whisker signals *in vivo*, the individual neurons in response to WS and OS were analyzed by intracellular recording. The responses of population neurons were recorded by LFP. The activities of network neurons and astrocytes were recorded by two-photon cell Ca^2+^ imaging.

**Figure 5:**
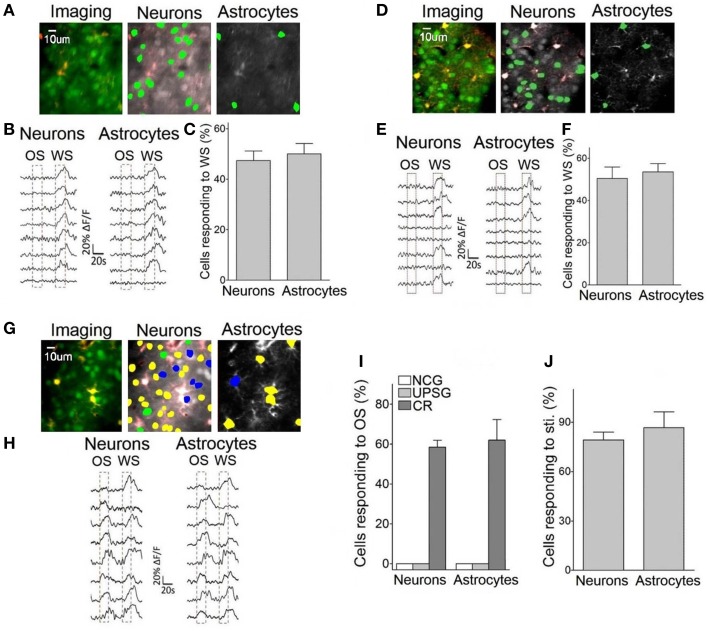
**Neurons and astrocytes in the barrel cortex respond to the odor-test after pairing OS and WS**. Cellular activities were detected by imaging Ca^2+^ signals under the two-photon microscope in the light anesthesia mice from NCG (*n* = 5), UPSG (*n* = 7), and CR-formation (*n* = 5), in which the astrocytes were labeled by SR101 (red). Panels **(A–C)** are from NCG mice, **(D–F)** are from UPSG, and **(G**–**J)** are from CR-formation. **(A)** Illustrates Ca^2+^ imaging (left panel, green for neurons, and red for astrocytes), neurons (middle) and astrocytes (right) in response to WS from a NCG mouse. **(B)** Illustrates Ca^2+^ signals from the neurons (left) and astrocytes (right) by giving OS and WS (red dash-line boxes) to a NCG mouse, in which they respond to WS only. **(C)** Shows the percentages of the neurons and astrocytes in response to WS from NCG mice (*n* = 5). **(D)** Illustrates Ca^2+^ imaging (left panel, green for neurons, and red for astrocytes), neurons (middle), and astrocytes (right) in response to WS from a UPSG mouse. **(E)** Illustrates Ca^2+^ signals from the neurons (left) and astrocytes (right) by giving OS and WS (red dash-line boxes) to a UPSG mouse, in which they respond to WS only. **(F)** Illustrates the percentages of the neurons and astrocytes in response to WS from UPSG mice (*n* = 7). **(G)** Shows Ca^2+^ imaging (left panel, green for neurons, and red for astrocytes), neurons (middle), and astrocytes (right) from a CR-formation mouse. The neurons and astrocytes responding to both OS and WS are labeled as yellow. Those responding to OS or WS only are labeled by blue or green. **(H)** Illustrate Ca^2+^ signals in the neurons (left) and astrocytes (right) responding to OS and WS from a CR-formation mouse. **(I)** Shows the portions of neurons (58.47 ± 7.8%) and astrocytes (62 ± 23.1%) in response to OS from CR-formation mice (dark-gray bars), compared with zero in NCG (white) and UPSG (light-gray). **(J)** The portions of the neurons and astrocytes in response to all stimuli from CR-formation mice (*n* = 5) are 79.3 ± 4.72 and 86.67 ± 9.72% of the OGB-detected cells, respectively.

Figure 5 illustrates Ca^2+^ imaging from the barrel cortical neurons and astrocytes in response to OS and WS. In NCG mice (*n* = 5), the neurons (green-labeled cells in middle panel of Figure 5A and traces in Figure 5B) and the astrocytes (green-labeled cells in Figure 5A right panel and traces in Figure 5B) respond to WS, but not OS. In total OGB-labeled cells, the neurons and astrocytes in response to WS are 47.4 ± 3.8 and 50 ± 4.12%, respectively (Figure 5C). Similarly, the neurons and astrocytes in UPSG mice respond to WS, but not OS (Figures 5D,E; *n* = 7). In OGB-labeled cells, the neurons and astrocytes in response to WS are 50.43 ± 5.4 and 53.52 ± 3.96%, respectively (Figure 5F). Except for these WS-responsive cells, a part of barrel cortical cells do not encode whisker sensation, which may be used for other physiological events, such as sensory plasticity and associative memory.

Interestingly, some neurons and astrocytes in the barrel cortices from CR-formation mice respond to WS and OS, respectively (yellow-labeled cells in Figure 5G, called as OS-/WS-responsive cells or CR cells), while some cells respond to OS (blue) or WS (green). Ca^2+^ signals in Figure 5H illustrate that the neurons and astrocytes respond to OS and WS, respectively. To the portions of the cells responding to OS (OS-/WS-responsive and OS-responsive cells) in the OGB-labeled cells, the neurons and astrocytes are 58.5 ± 7.8 and 62.1 ± 23.1% from CR-formation mice (*n* = 5; dark-gray bars in Figure 5I), compared with zero in NCG (whites) and UPSG mice (light-grays). A substantial amount of barrel cortical neurons and astrocytes become to encode the acquired odor signal besides innate whisker signal after their association, i.e., the associated signals are stored in the individual neurons and astrocytes that are called as associative memory cells or conditioned reflex cells (CR cells). Moreover, the neurons and astrocytes in response to all stimuli are 79.3 ± 4.7 and 86.7 ± 9.7% in total detected cells (Figure 5J). Compared with the portion of WS-responsive cells in NCG and UPSG mice (Figures 5C,F), the cells are recruited in response to OS from CR-formation mice. These data indicate that some inactive cells in NCG and UPSG mice (Figure 5C) are recruited or refined to be associative memory cells for the odor and whisker signals.

**Figure 6:**
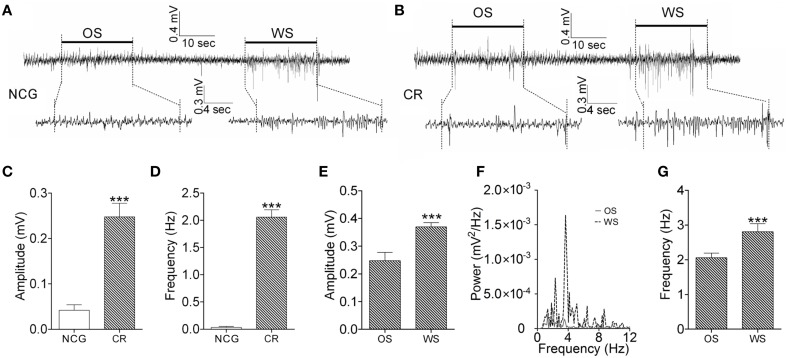
**The activity patterns of barrel cortical neurons in response to OS and WS from CR-formation mice and controls**. Neuronal activities were recorded by LFP *in vivo*. **(A)** Top trace shows that the neurons in the barrel cortex from a NCG mouse do not respond to OS (left horizontal bar), but respond to WS (right). Calibration bars for this trace are 0.4 mV and 10 s. Bottom traces illustarte the expanded waveforms from the fragments of no response to OS (left) and of response to WS (right). Calibration bars for these traces are 0.3 mV and 4 s. **(B)** Top trace shows that the neurons in the barrel cortex from a CR-formation mouse respond to OS (left horizontal bar) and WS (right). Calibration bars for this trace are 0.4 mV and 10 s. Bottom traces show the expanded waveforms from the fragments of responses to OS (left) and WS (right). LFP amplitude and frequency appear different. Calibration bars for these traces are 0.3 mV and 4 s. **(C,D)** Show LFP amplitudes **(C)** and frequencies **(D)** in response to OS, which are recorded in the barrel cortex from NCG mice (white bar; *n* = 9) and CR-formation mice (gray; *p* < 0.001, *n* = 9; One-Way ANOVA). **(E)** Shows LFP amplitudes recorded from the barrel cortex of CR-formation mice in response to WS and OS (*p* < 0.001, *n* = 9; One-Way ANOVA). **(F)** Illustrates the power-spectrum of LFP frequency in the barrel cortex of CR-formation mice in response to WS (dash line) and OS (solid line). **(G)** Shows LFP frequency recorded in the barrel cortex of CR-formation mice in response to WS and OS (*p* < 0.001, *n* = 9; One-Way ANOVA). ^***^p < 0.001.

We also examined the recruitment of barrel cortical neurons to encode odor and whisker signals by recording LFP in barrel cortices. The neurons in a CR-formation mouse respond to both WS and OS (Figure 6B), compared with the neurons in a control mouse that do not respond to OS (Figure 6A). Figures 6C,D illustrates the averaged LFP amplitude and frequency in response to OS from CR-formation mice (gray bars, *n* = 9) and control mice (whites; *p* < 0.001, *n* = 9; One-Way ANOVA). The barrel cortical neurons in CR-formation mice are recruited to encode the acquired odor signal alongside innate whisker signal.

The neurons and astrocytes in the barrel cortices become encoding odor and whisker signals after their association. How do the CR cells recognize these associated signals to be different in their retrievals? We hypothesize that the recognition to whisker and odor signals may be fulfilled by the process that CR cells encode these signals with different activity patterns.

### A population of neurons in the barrel cortex recognizes associative signals by activity patterns

We analyzed the responses of the barrel cortical neurons to WS and OS by LFP recording and two-photon cell imaging *in vivo* from CR-formation mice. The patterns of these neurons in responses to OS and WS appear different (Figure 6B). LFP amplitudes are 0.25 ± 0.03 mV in response to OS and 0.37 ± 0.02 mV to WS (Figure 6E). LFP frequencies are 2.1 ± 0.15 Hz in response to OS and 2.8 ± 0.3 Hz to WS (Figures 6F,G). LFP amplitude and frequency in barrel cortical neurons from CR-formation mice are different in response to odor and whisker signals (*p* < 0.001, *n* = 9; One-Way ANOVA). This result indicates that a population of neurons in barrel cortices is able to recognize the odor and whisker signals.

**Figure 7:**
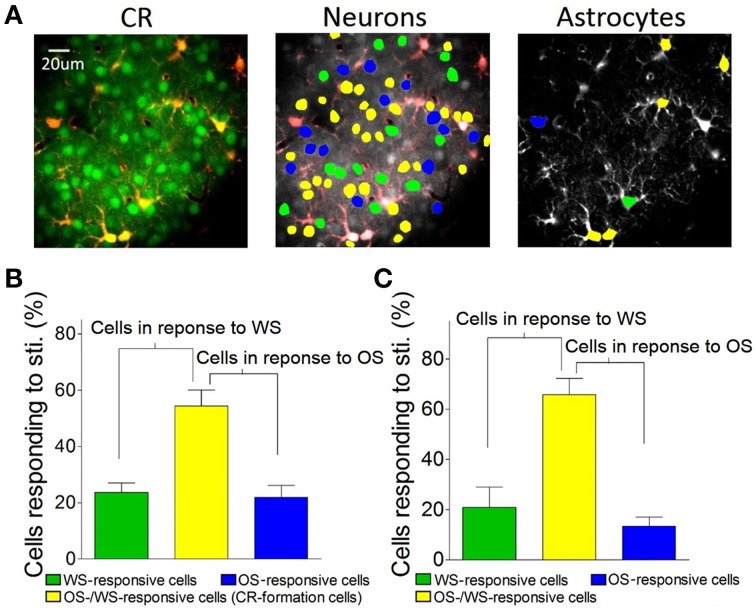
**The barrel cortex recognizes whisker and olfaction inputs via organizing different neurons in their responses. (A)** Shows Ca^2+^ imaging in neurons (greens in left panel) and astrocytes (red). The neurons (middle) and astrocytes (right) in response to both OS and WS are labeled as yellow, OS only as blue, or WS only as green. **(B)** Shows the percentages of WS-responsive neurons (green bar), OS/WS-responsive neurons (yellow), and OS-responsive neurons (blue) from CR-formation mice (*n* = 5). **(C)** Illustrates the percentages of WS-responsive astrocytes (green bar), OS/WS-responsive astrocytes (yellow), and OS-responsive astrocytes (blue) from CR-formation mice (*n* = 5). WS-responsive and OS/WS-responsive cells work together to recognize whisker signal. OS-responsive and OS/WS-responsive cells work together to recognize odor signal.

In terms of the role of barrel cortical neurons and astrocytes in recognizing the associated signals, two-photon cell Ca^2+^ imaging shows OS-/WS-responsive cells (yellow-labeled cells in the middle panel of Figure 7A), OS-responsive cells (blue) and WS-responsive cells (green). In total neurons of responding to stimulations (Figure 5J), the portions of WS-responsive neurons, OS-responsive neurons, and OS-/WS-responsive neurons are 23.7 ± 7.5, 21.9 ± 9.6, and 54.4 ± 12.8%, respectively (*n* = 5 mice, Figure 7B). The portions of WS-responsive, OS-responsive, and OS-/WS-responsive astrocytes are 21 ± 18.2, 13.4 ± 8.2, and 65.6 ± 14.3%, respectively (Figure 7C). These results indicate that WS-responsive cells and OS-/WS-responsive cells work together to encode the whisker signal, but OS-responsive and OS-/WS-responsive cells work together to encode the odor signal. The responsive neurons or astrocytes in the barrel cortices are organized into two populations to distinguish the input signals from either whiskers or olfaction.

### Individual neurons in the barrel cortex recognize associative signals during memory retrieval

The roles of individual CR cells in distinguishing WS and OS were studied in the barrel cortex *in vivo*. In two-photon imaging of cells, their spatial patterns were measured by responsive strength and their synchronies were analyzed by cells' cross-correlations (Zhao et al., 2012). In intracellular recording, the activity patterns in the individual neurons were analyzed as the frequencies of the neuronal spikes and synaptic activity.

**Figure 8:**
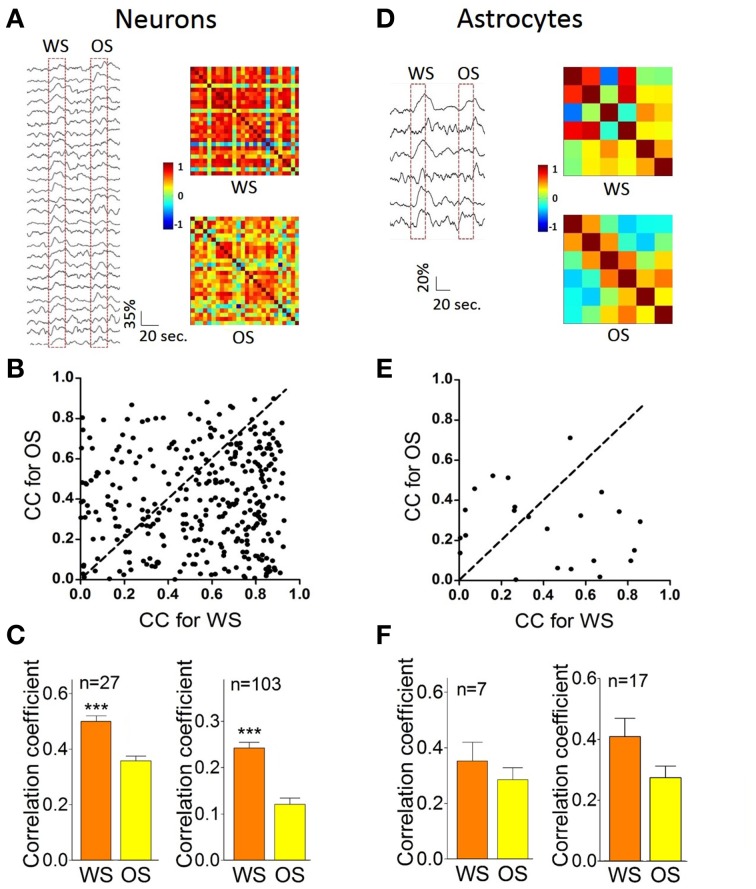
**The individual CR cells in the barrel cortex recognize odor and whisker input signals by coding their distinct activity patterns. (A–C)** Present the data from neuron pairs and **(D–F)** show the data from astrocyte-pairs. **(A)** Shows temporal patterns from CR neurons in response to WS (right-top panel) and OS (right-bottom one) from an experiment, in which the responses of 27 neurons to WS and OS (red dash-line boxes) are presented in left panel. Calibration bars are 35% changes and 20 s. Each pixel in matrices denotes the peak value of correlation coefficient for a pair of cells. Dark-red pixels show the best cross-correlation (synchrony), or vice versa. **(B)** Illustrates correlation coefficients (CC) for OS vs. CC for WS in this example. A dash-line in 45° denotes equal values in CC for OS vs. CC for WS. The different activity synchronies are seen among most neuron pairs in response to WS and OS. **(C)** Shows statistical comparisons in CC peak values for the neurons in response to WS and OS from this experiment (left panel; *p* < 0.001, *n* = 27; paired *t*-test), and those for the neurons in response to WS and OS from five experiments (right; *p* < 0.001, *n* = 103; paired *t*-test). **(D)** Shows temporal patterns from CR astrocytes in response to WS (right-top panel) and OS (right-bottom) from an experiment, in which the responses of the astrocytes to WS and OS are showed in left panel. Calibration bars are 20% changes and 20 s. **(E)** Shows CC for OS vs. CC for WS in this example. Dash-line in 45° indicates equal values in CC for OS vs. CC for WS, indicating the different activity synchrony among most astrocyte pairs in response to WS and OS. **(F)** Illustrates statistical comparisons in CC peak values for the astrocytes in response to WS and OS from this experiment (left panel; *p* = 0.35, *n* = 7; paired *t*-test), and those for the astrocytes in response to WS and OS from five experiments (right; *p* = 0.1, *n* = 17; paired *t*-test). ^***^p < 0.001.

Figure 8 illustrates the temporal patterns of CR neurons and astrocytes in response to WS and OS. Each pixel in the matrices presents peak cross-correlation for a pair of neurons (right panels in Figure 8A) or a pair of astrocytes (right panels in Figure 8D) in response to WS (top) and OS (bottom) from an experiment. Dark-red pixels denote the best cross-correlation, or vice versa. The plot in Figure 8B shows correlation coefficients (CC) for responding to OS vs. CC to WS, indicating that the most neuron pairs possess different activity synchronies in response to WS and OS. Correlation coefficients for CR neurons (*n* = 27) from a mouse are 0.5 ± 0.036 in response to WS and 0.36 ± 0.03 to OS (left panel in Figure 8C; *p* < 0.0001; paired *t*-test) from the experiment in Figures 8A,B. Correlation coefficients for CR neurons (*n* = 103) from five mice are 0.43 ± 0.026 in response to WS and 0.35 ± 0.037 to OS (right panel in Figure 8C; *p* < 0.0001; paired *t*-test). Figure 8F illustrates correlation coefficients for CR astrocytes in response to WS (0.41 ± 0.28) and to OS (0.28 ± 0.18, *p* = 0.1; paired *t*-test; right panel) from five mice. The results support a hypothesis that CR neurons recognize the whisker and odor signals by synchronizing their activities.

**Figure 9:**
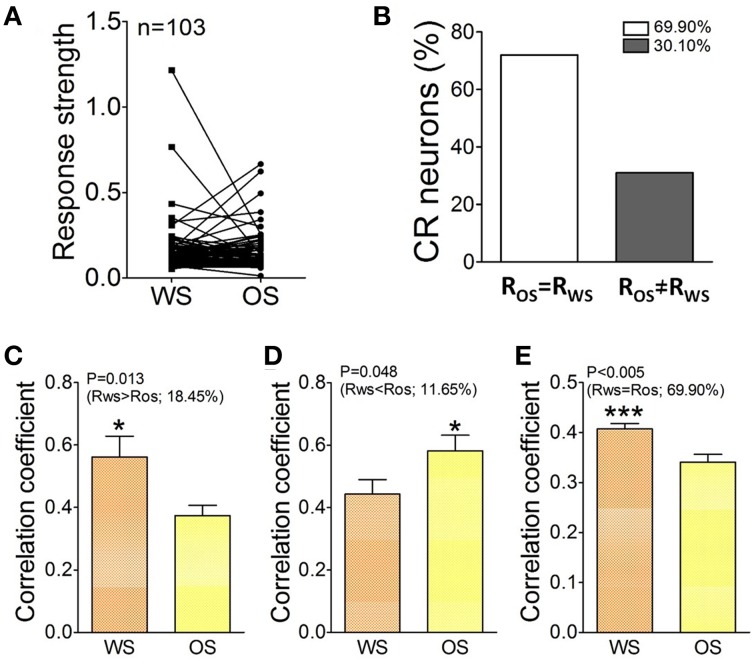
**The activity level and cross-correlation of barrel cortical CR neurons in response to OS and WS. (A)** Activity levels from CR neurons in response to WS and OS are different (*n* = 103 neurons). **(B)** Shows the percentages of CR neurons with equal strength (R_WS_ = R_OS_, white bar, 69.9%) vs. distinct strength (R_WS_≠ R_OS_, gray, 30.1%). **(C)** Illustrates correlation coefficients for 18.45% CR neurons with R_WS_ > R_OS_ in response to WS (orange bar) and OS (yellow; *p* = 0.013; paired *t*-test). **(D)** Shows correlation coefficients for 11.65% CR neurons with R_WS_ < R_OS_ in response to WS (orange bar) and OS (yellow, *p* = 0.04; paired *t*-test). **(E)** Shows correlation coefficients for 69.9% CR neurons with R_WS_ = R_OS_ in response to WS (orange bar) and OS (yellow, *p* = 0.005; paired *t*-test). The recognition of barrel cortical neurons to WS and OS by encoding their different activity synchronies. ^*^p < 0.05; ^***^p < 0.001.

In terms of activity levels, Figures 9A,B shows Ca^2+^ signals from CR neurons in responses to OS and WS. If the difference of their responses to WS vs. OS is above 2.5 times of standard deviation of averaged values (i.e., R_WS_≠ R_*OS*_), we assume that they are able to distinguish odor and whisker signals. 30.1% neurons respond to WS and OS with different strengths (Figure 9B). The recognition of whisker and odor signals may also be fulfilled by setting the activity levels in some CR neurons. Furthermore, the changes of neuronal responses to WS and OS in the cross-correlation and activity level appear parallel. Correlation coefficients for the neurons with R_WS_ > R_OS_ are 0.56 ± 0.043 in response to WS and 0.374 ± 0.022 to OS (Figure 9C; *p* = 0.013, paired *t*-test). Correlation coefficients for the neurons with R_WS_ < R_*OS*_ are 0.44 ± 0.023 in response to WS and 0.59 ± 0.025 to OS (Figure 9D; *p* = 0.04; paired *t*-test). Correlation coefficients for the neurons with R_WS_ = R_*OS*_ are 0.41 ± 0.035 in response to WS and 0.34 ± 0.024 to OS (Figure 9E; *p* < 0.005; paired *t*-test). Thus, the CR neurons in the barrel cortex recognize whisker and odor signals by changing their functional connection and activity level.

**Figure 10:**
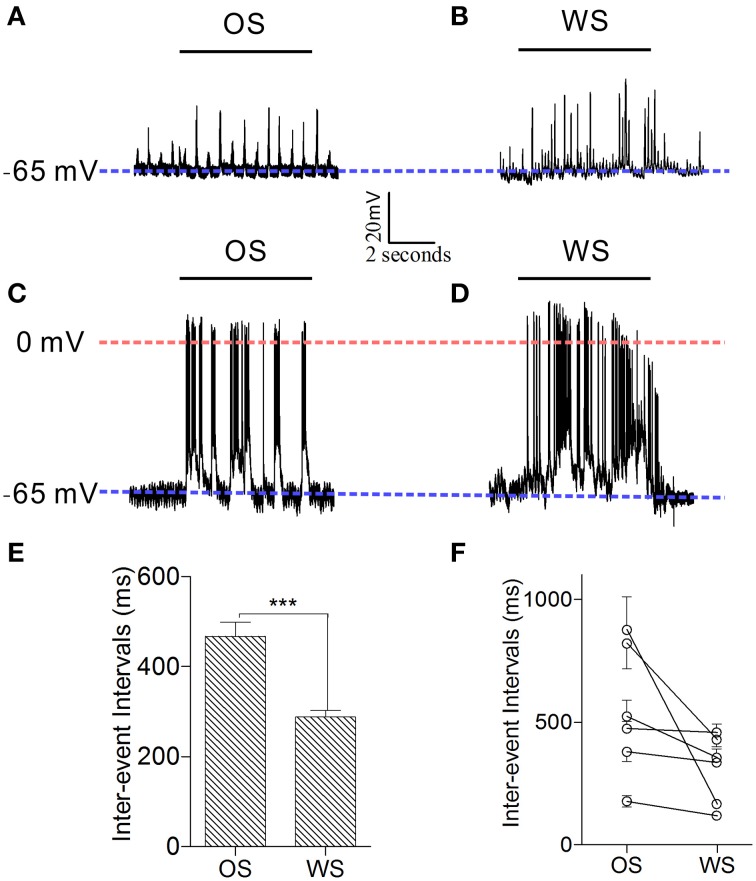
**Individual neurons in the barrel cortex can recognize OS and WS from CR-formation mice by encoding their activity patterns**. Neuronal activities were recorded by intracellular recording. **(A,B)** Show that a neuron responds to OS (horizontal bar in **A**) and WS (horizontal bar in **B**) with different synaptic integrated events. Blue dash-line illustrates resting membrane potential (−65 mV) for this neuron. **(C,D)** Illustrate that a neuron responds to OS (horizontal bar in **C**) and WS (horizontal bar in **D**) with different spike patterns. Blue dash-line shows resting membrane potential (−65 mV) for this neuron, and red dash-line shows a zero membrane potential for indicating the overshot of action potentials. Calibration bars are 20 mV/2 s. **(E)** Shows the averaged inter-event intervals from neurons in response to OS and WS (*n* = 6, *p* < 0.001; One-Way ANOVA). **(F)** Shows inter-event intervals from each of six neurons that respond to OS and WS. ^***^p < 0.001.

In order to confirm that individual neurons were able to recognize associative signals, we further recorded barrel cortical CR neurons intracellularly and analyzed the patterns of their responses to OS and WS. Figures 10A,B shows an example of recording synaptic integrated potentials in response to OS and WS. Figures 10C,D shows an experiment of recording spike bursts in response to OS and WS. Statistical analyses in inter-event intervals (Figures 10E,F) show that the activity patterns in CR neurons are distinct in response to WS and OS (*p* < 0.001, *n* = 6; One-Way ANOVA). The result is consistent with that from two-photon cell imaging. The individual neurons in the barrel cortex memorize associative whisker and odor signals, as well as recognize their differences by encoding different responsive patterns in signal retrieval.

## Discussion

**Figure 11:**
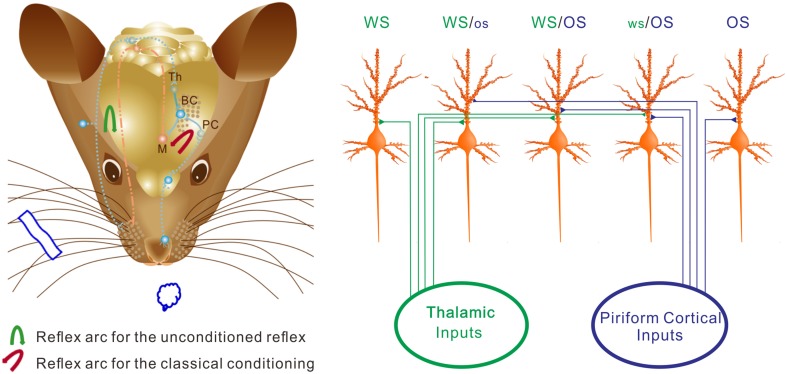
**The neurons in the barrel cortex are reorganized after associative learning. (Left)** Shows a mouse brain including reflex arcs for whisker-induced whisker motion (unconditioned reflex; blue arrow) and odorant-induced whisker motion (cross-modal reflex; red arrow) after WS/OS-pairing. Associative learning induces the connection between the barrel and piriform cortices, which allows the formation of cross-modal reflex. Afferent pathways (blue dot lines) and efferent pathway (red dash-dot) in the reflex arc are showed under the cerebral cortex. The connections from the barrel cortex (BC) to piriform cortex (PC) and from BC to the motor cortex (M) in the center of the reflex arc are presented as solid blue lines. **(Right)** Shows barrel cortical neurons that are recruited and refined as WS/OS-responsive neurons (three neurons in the middle), receive the axonal innervations from the thalamus and piriform cortex, and encode WS and OS, i.e., associative memory neurons. Left-middle neuron receives the thalamic input and responds to WS dominantly. Right-middle neuron receives the piriform cortical input and responds to OS dominantly. Middle neuron receives the thalamic and piriform inputs and responds to WS and OS equally. Moreover, some neurons receive thalamic input naturally and respond to WS only **(left)**, whereas some neurons receive piriform cortical input after associative learning and respond to OS only **(right)**. These OS-responsive cells for cross-modal memory fall into the category of new signal memory cells. Based on the different responses of individual neurons to WS and OS and the different organizations of responsive neurons, the barrel cortex becomes able to fulfill the associative storages and distinguishable retrievals of the newly acquired odor signal and the innate whisker signal.

We study the recruitment of the cortical neurons and astrocytes for the storage and retrieval of the associated signals in a new mouse model of conditioned reflex. A simultaneous pairing of whisker and odor stimuli leads to odorant-induced whisker signal recall and whisker motion (Figure 1). An associative activation of the barrel and piriform cortices induces their synaptic connections (Figure 3). The afferent pathways are convergent into the sensory cortices and share the common efferent pathway in their reflex arcs (Figure 11) for co-expressing native reflex (whisker-induced whisker motion) and conditioned reflex (odorant-induced whisker motion). With the convergence of sensory pathways into the barrel cortex, a substantial amount of the neurons, and astrocytes are recruited to encode new odor signal and innate whisker signal for their associative storages (Figures 5–7). The union of these associated signals through their respective pathways (Figure 11) synaptically onto individual cells recruits associative memory cells, such that the associated signals retrieve each other. Moreover, the associative memory cells express different or similar patterns in response to the associated signals (Figures 6–10). Some cells by computing the different codes distinguish the differences of the associated signals, and others by identical patterns signify the historical association of these signals. This working principle for associative memory is granted by the observation that the cross-modal reflex and associative memory cells are present for multiple signals.

In terms of physiological impact for individual neurons to encode associative signals, the memory of multiple signals in each individual neuron saves the number of neurons needed for information storage, or expands memory capacity in the brain. The recognition of the associated signals by each neuron allows the precise memory retrieval. This principle of designing neurons as efficient memory units is useful to build electronic elements for information storage. On the other hand, the memory of associative signals in multiple neurons prevents loss of memorized signals. Network neurons to recognize signal sources may help to retrieve a specific stored signal by a spectrum of cues similar to its associative signals because the cerebral neurons possess variable excitability (Wang et al., 2008; Zhang et al., 2012) and response strengths to the given cues (Figures 9, 10).

In information retrieval, the cues are needed to access the neurons that encode memory (Fletcher et al., 1996; Gandhi, 2001; Otten, 2007; Winters et al., 2008). The brain appears aware of whether a given cue is similar to one of the associated signals. In the case of their similarity, the memory units respond to the cue and this signal, i.e., this cue retrieves it. The associative memory cells that store two associated signals allow another of the associated signals to be retrieved. Some associative memory cells demonstrate similar activity patterns in response to innate and new signals (Figure 9B), they encode these associated signals and the cues as similar events or associated events. Others respond to new and innate signals with different activity patterns (Figures 8–10), so that these cells distinguish the signals from different sources during their retrievals. The network neurons can also distinguish signal sources (Figures 6, 7). This process may be one of mechanisms that the cues with different natures from the associated signals cannot retrieve these signals, i.e., retrieval specificity (Figure 4C). The involvement of multiple processes in signal recognition makes information retrieval to be efficient and precise for sorting useful messages and managing well-organized behaviors in the life.

In the studies of associative learning, the animal models of conditioned reflexes are used, such as eyeblink-conditioning (Burhans et al., 2008; Woodruff-Pak and Disterhoft, 2008; Bracha et al., 2009) and fear-conditioning in the rodents (Davis et al., 1993; Reijmers et al., 2007; Maren, 2008; Perkowski and Murphy, 2011), as well as withdrawal reflex in Aplysia (Hawkins, 1984; Glanzman, 1995; Lechner et al., 2000). In these studies, the motor-related brain areas and motor neurons presumably process the information storage. In our mouse model of odorant-induced whisker motion, the barrel cortex is critical for conditioned reflex (Figure 4), and their neurons work for the primary process of information storage and retrieval (Figures 5–10). These differences can be interpreted by a fact that the expression of conditioned reflex is fulfilled by the neuronal circuits from the sensory cortices to the motor cortices. The stimulus to any of these areas in neural circuits can evoke conditioned reflex (Ehrlich et al., 2009; Pape and Pare, 2010; Liu et al., 2012; Li et al., 2013; Xu and Südhof, 2013). In this regard, the sensory cortices in neural circuits are still the primary locations for information storage and retrieval. The motor-related neurons are the common pathway to encode motion orders for sending these orders out, and have low capacities to store the signals. On the other hand, the sensory cortical neurons to integrate and encode the associated signals for their storage and retrieval will designate signal specificity and expand memory volume. This design is the efficient division of labors in the mammalian's brain during evolution.

Associative learning by pairing whisker and odor signals induces the mutual innervation between the barrel and piriform cortices (Figure 3), which grants their functional communications for the storage and retrieval of the associated signals and cross-modal memory (Wang et al., 2014). This reciprocal cross-modal memory makes the terms of the unconditioned and conditioned stimuli not being present, i.e., either whisker signal or odorant signal will induce cross-modal reflex. In addition, the barrel and piriform cortices connect in cross-modal plasticity, which upregulates the functions of their partner cortices (Ye et al., 2012). New connections among cortical areas are basically for their physiological coordination and the mutual use in their reflex arcs (Figure 11) to fulfill reciprocal cross-modal reflexes (Wang et al., 2014) and to prevent arcs' deficit after loss of their uses. In this regard, associative learning facilitates the establishment of more connections among cortical function units and the formation of more cross-modal memories. Moreover, our data, which the co-activation of the sensory cortices with different modalities leads to their connections, upgrades Hebb's hypothesis that groups of repeatedly co-activated cells become wired (Hebb, 1949; Lansner, 2009).

In terms of the recruitment of the barrel cortical neurons and astrocytes to be associative memory cells, our results indicate that its fulfillment is initiated by associating the odorant signal from the piriform cortex and the whisker signal from the thalamus, but neither the whisker signal or odor signal alone. This indication is based on the facts that the number of responsive cells increases from 50% (WS-responsive cells) in NCG or UPSG mice to 80% (WS-responsive, WS/OS-responsive, and OS-responsive cells) in CR-formation mice (Figure 5), and there is no statistical difference in the percentages of WS-responsive cells between NCG and UPSG mice. Moreover, for barrel cortical cells innervated by new connection from the piriform cortex, our results indicate that OS-responsive cells are 55% of total responsive cells (Figure 5H). That the portion of OS-responsive cells is greater than the recruited cells (30%) indicates that the axons from the piriform cortex innervate onto WS-responsive and WS non-responsive cells in the barrel cortex. The recruitment of inactive neurons and the refinement of WS-responsive neurons are involved in the storage and retrieval of the odor signal newly to the barrel cortex.

Why barrel cortical neurons can distinguish whisker and olfactory signals is based on a possibility that associative memory cells receive the synaptic inputs, which carry the associated signals, with distinct strength. For instance, the averaged strength of the barrel cortical neurons is higher in response to whisker signal than odor one (Figures 6, 8–10). The weight of synaptic inputs to most barrel cortical neurons is higher from the thalamus than the piriform cortex (Figure 3A). Some barrel cortical neurons show higher strength and synchrony (Figures 7, 8) in response to whisker signal than odor one, or vice versa. How are whisker-dominant and odor-dominant neurons in the barrel cortex are formed? The co-activations of the barrel cortex and the piriform cortex initiate axonal growth toward each other (Figure 3). The axons from the piriform cortex to the barrel cortex meet WS-responsive neurons, but these new axons may not be able to compete over natural axonal innervations from the thalamus. WS-responsive neurons become encoding WS and OS, but whisker-dominant neurons. The axons from the piriform cortex that are not taken by WS-responsive neurons turn toward WS non-responsive neurons and compete with the thalamic axons. These WS non-responsive neurons are recruited and refined to encode both OS and WS, and some of them become odor-dominant neurons (Figure 11).

In our study, each of our questions is examined by two approaches. The role of neural networks in associative memory is investigated by LFP and two-photon cell imaging. The roles of individual neurons in signal storage and retrieval are studied by two-photon cell imaging and intracellular recording. Synaptic connections between the barrel and piriform cortices are confirmed by neural tracing and electrophysiology. The consistent results by multiple approaches strengthen our conclusion that both neural networks and single neurons play critical roles in the storage and retrieval of the associated signals. Importantly, our new mouse model of cross-modal reflex will assist to reveal the working principles of associative memory cells based on WS-/OS-responsive cells for the distinguishable storage and retrieval of the associated signals, as well as of new memory cells based on OS-responsive cells for encoding novel signals.

## Author contributions

DW, JZ, ZG, NC, BW, CC, YL, and JF contribute to experiments and data analyses. WL contributes to the production of the digitized MSMS. ZL contributes to the software writing for cross-correlation analysis. JW contributes to project design and paper writing.

### Conflict of interest statement

The authors declare that the research was conducted in the absence of any commercial or financial relationships that could be construed as a potential conflict of interest.

## Abbreviations

CR: conditioning response and conditioned reflex
WS: whisker stimulus
OS: odor stimulus
LFP: local field potential
PSG: paired stimulus group
UPSG, unpaired stimulus group, NCG: naïve
control group.
