# Supplementary material for: Neurons in the barrel cortex turn into processing whisker and odor signals: a cellular mechanism for the storage and retrieval of associative signals
Source: Front Cell Neurosci. 2015 Aug 21;9:320. doi: 10.3389/fncel.2015.00320 (PMC4543922; doi:10.3389/fncel.2015.00320)
Supplement: Supplementary file 3 [file DataSheet1.DOC]

**Submission to: Frontiers in Cellular Neuroscience**

**Neurons in the barrel cortex turn into processing whisker and odor signals: a cellular mechanism for the storage and retrieval of associative signals**

**Dangui Wang^1#^, Jun Zhao^1,2#^, Zilong Gao^1,2#^, Na Chen^1^, Bo Wen^1^, Wei Lu^1^, Zhuofan Lei^1^, Changfeng Chen^3^, Yahui Liu^3^, Jing Feng^1^ and Jin-Hui Wang^1,2,3^***

***1) State Key Lab of Brain and Cognitive Science, Institute of Biophysics, Chinese Academy of Sciences, Beijing China 100101; 2) University of Chinese Academy of Sciences, Beijing China 100049; 3) Department of Physiology, Bengbu Medical College, Bengbu Anhui 233000***

Running title: Storage and retrieval of associative signals in neurons

Key words: conditioned reflex in mouse, learning, memory, neuron, astrocyte, barrel cortex, whisker and olfaction

Word counts: abstract, 241; text, 11877

# Wang D, Zhao J and Gao Z contribute to this work equally

Abbreviations: CR, conditioning response and conditioned reflex, WS, whisker stimulus; OS, odor stimulus; LFP, local field potential; PSG, paired stimulus group; UPSG, unpaired stimulus group, NCG, naïve control group.

**Corresponding author:**

Jin-Hui Wang, Ph.D. & MD

The Institute of Biophysics, the Chinese Academy of Sciences

15 Datun Road, Chaoyang District

Beijing China 100101

[jhw@sun5.ibp.ac.cn](mailto:jhw@sun5.ibp.ac.cn); 86-10-64888472

## Figure S1 Butyl acetate test-pulse evokes the responses of nerve cells in mouse olfactory bulb under the two-photon microscopy. A) shows Ca^2+^ imaging in the neurons (green in left panel) and astrocytes (red) in olfactory bulb under the stimulation of butyl acetate pulses, in which the neurons activated by butyl acetate are labeled by yellow (right panel). B) Butyl acetate pulse toward the noses induces the changes of Ca^2+^ signal in the neurons of olfactory bulb, which are the part of yellow-labeled neurons in top panel.

**
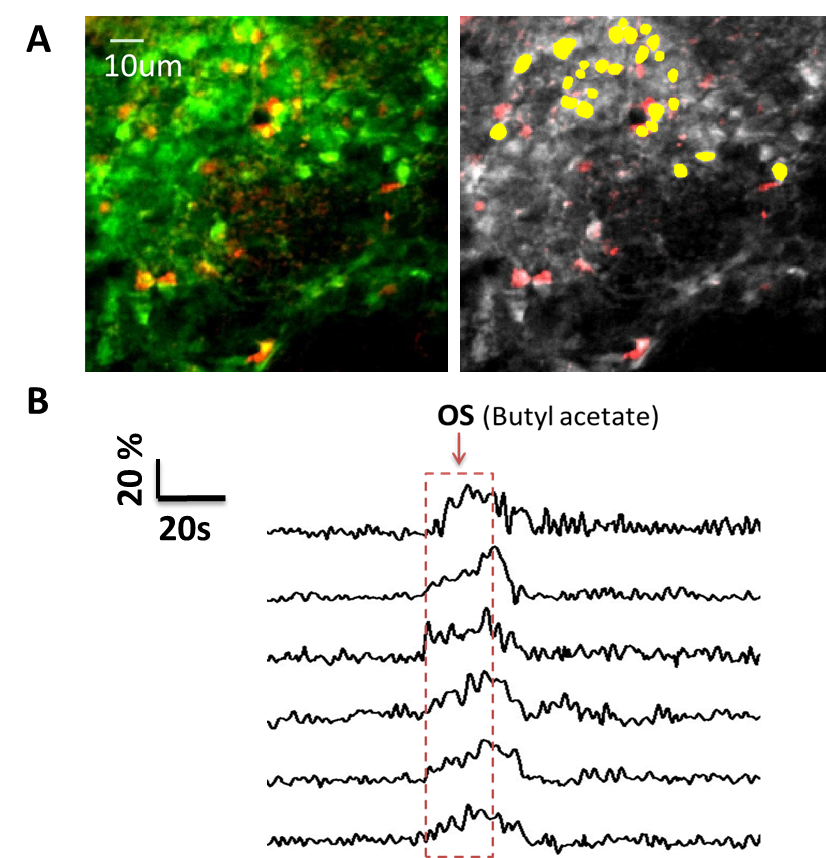
**

##

## Video one: The training paradigm by pairing whisker stimulus (WS) and odor stimulus (OS)

**Video two:** Odorant-induced whisker motion tested by odorant stimulus (butyl acetate)
